# Characterization of Hepatitis C Virus Recombination in Cameroon by Use of Nonspecific Next-Generation Sequencing

**DOI:** 10.1128/JCM.00483-15

**Published:** 2015-09-16

**Authors:** James C. Iles, Richard Njouom, Yacouba Foupouapouognigni, David Bonsall, Rory Bowden, Amy Trebes, Paolo Piazza, Ellie Barnes, Jacques Pépin, Paul Klenerman, Oliver G. Pybus

**Affiliations:** a Department of Zoology, University of Oxford, Oxford, United Kingdom; b Service de Virologie, Centre Pasteur du Cameroun, Yaounde, Cameroon; c Department of Microbiology and Infectious Diseases, Université de Sherbrooke, Sherbrooke, Canada; d Peter Medawar Building for Pathogen Research, University of Oxford, Oxford, United Kingdom; e Wellcome Trust Centre for Human Genetics, Oxford University, Oxford, United Kingdom

## Abstract

The importance of recombination in the evolution and genetic diversity of the hepatitis C virus (HCV) is currently uncertain. Only a small number of intergenotypic recombinants have been identified so far, and each has core and envelope genes classified as belonging to genotype 2. Here, we investigated two putative genotype 4/1 recombinants from southern Cameroon using a number of approaches, including standard Sanger sequencing, genotype-specific PCR amplification, and non-HCV-specific Illumina RNA sequencing (RNA-seq). Recombination between genotypes 1 and 4 was confirmed in both samples, and the parental lineages of each recombinant belong to HCV subtypes that are cocirculating at a high prevalence in Cameroon. Using the RNA-seq approach, we obtained a complete genome for one sample, which contained a recombination breakpoint at the E2/P7 gene junction. We developed and applied a new method, called Deep SimPlot, which can be used to visualize and identify viral recombination directly from the short sequence reads created by next-generation sequencing in conjunction with a consensus sequence.

## INTRODUCTION

Hepatitis C virus (HCV) is a globally prevalent and genetically diverse virus that infects approximately 170 million people worldwide (1). HCV is classified into seven genotypes (1 to 7), with an average 35% nucleotide divergence between strains belonging to different genotypes (2). All genotypes except 5 and 7 are subdivided into numerous subtypes (1a, 1b, 1c, 2a, 2b, etc.), and intrasubtypic nucleotide divergence is typically <15%. Despite this large genetic diversity, there has been comparatively little evidence that recombination plays a significant role in HCV evolution, particularly in comparison to HIV-1 (3). Several naturally occurring HCV recombinants have been reported, and in 17 cases, a complete virus genome has been obtained (summarized in Table 1), although the methods used to confirm a mosaic genome structure varied among the studies. While breakpoints for intragenotypic recombinants are seen throughout the viral genome, the breakpoints of intergenotypic recombinants are seen only in the nonstructural 2 (NS2) gene or near the NS2/NS3 boundary (Table 1). Additionally, many intergenotypic recombinants are classified as genotype 2 at the 5′ end of the genome, while the genotype classification of the 3′ end varies. With one exception (4), all the HCV recombinants in Table 1 have only one reported breakpoint. In contrast, HIV-1 recombinants often have multiple breakpoints, and secondary recombination between HIV-1 recombinant strains has led to increasingly complex patterns of genome inheritance (5).

**TABLE 1:** Details of HCV recombinants proposed in the literature

Genotype or subtype	Location of estimated recombination breakpoint(s)		Source
	Gene(s)	Nucleotide position(s)^*a*^	
1a/1c	E1, E2	1407, 2050	10
1a/1c	Core, E1, E2, NS2, NS3	801, 1261, 2181, 3041, 3781	4
1b/1a	NS5B	8320	47
1b/1a	Core	387	48
**2/5**^*b*^	NS2/NS3	3420–3440	49
**2b/1a**	NS2/NS3	3405–3416	50
**2b/1b**	NS2/NS3	3443	51
**2b/1b**	NS2/NS3	3399	52
**2b/1b**	NS2	3298–3305	9
**2b/6w**	NS2/NS3	3429	53
**2k/1b**	NS2/NS3	3175	6
4d/4a	Between E2 and NS5A	Unknown	54
6a/6o	NS5B	8345	11
6e/6h	NS5B	8356	11
6e/6o	NS5B	8358	11
6n/6o	NS5B	8372	11

a Nucleotide positions are relative to isolate H77.

b Entries in bold are intergenotypic recombinants.

HCV recombinants are classified according to their potential epidemiological significance. Unique recombinant forms (URFs) are those found in one patient only (or in closely linked patients), while circulating recombinant forms (CRFs) are those found in multiple patients. Only one HCV CRF has been discovered to date: a mosaic of subtypes 2k/1b that was initially discovered in 2002 in injecting drug users in St. Petersburg, Russia (6, 7, 8). Although the 2b/1b recombinant reported by Hoshino et al. (9) was detected in two different patients, they attended the same clinic and thus may be epidemiologically linked. The deficit of HCV CRFs implies that the epidemiological significance of recombination in HCV is low. Alternatively, the prevalence of HCV recombinant forms may be underestimated due to the use of genotyping methods that are unlikely to detect recombination, such as serotyping and single-locus sequencing (10).

A better understanding of recombination in HCV is of practical importance and evolutionary interest. In general, recombination accelerates viral adaptation by bringing together multiple advantageous mutations that may provide drug resistance or escape from the host immune system (11). The development of direct-acting anti-HCV therapies (12) potentially increases the clinical importance of recombination for HCV. However, current direct-acting anti-HCV drugs target only the nonstructural proteins of the virus, so it seems unlikely that intergenotypic recombination will generate multiple resistances, as intergenotypic recombination tends to occur between the structural and nonstructural genome regions (see Table 1). Thus, any potential impact of recombination on anti-HCV therapy will likely arise from intragenotypic recombination. There is already some evidence that the 2k/1b CRF is less responsive to antiviral therapy than strains composed solely of genotype 2 or 3 (7).

The mechanism by which HCV recombination occurs *in vivo* is currently unknown, as superinfection exclusion via CD81 downregulation is thought to make infection of a single cell by multiple HCV strains unlikely (13). A common model of recombination in RNA viruses is the copy choice mechanism, in which the viral RNA-dependent RNA polymerase (RdRp) switches during replication from a donor template RNA to an acceptor template RNA, without releasing the nascent strand being produced (14, 15). The frequency of switching may depend on conserved sequences and RNA secondary structures in both strands. A second mechanism of recombination is RdRp-independent breakage and rejoining, in which two RNA molecules of different strains are broken (via restriction enzymes or mechanical stress) and subsequently fused by self-ligation or cellular ligases (3). This mechanism has been shown to create homologous and nonhomologous recombinants in the same virus family as HCV, but the action of this mechanism in HCV has yet to be shown (16).

A common method for detecting recombination in HCV is to amplify and sequence the sample in two genomic regions and estimate the phylogenies of the sample and reference sequences for each location. If recombination has occurred, the isolate will be associated with different reference genomes at each region, and statistical support can be assessed with phylogenetic bootstrap scores (17). While this is a simple method, it is also vulnerable to false-positive results. If the patient is coinfected with two HCV strains, PCR conditions and primers might favor the amplification of different strains in the two genome regions (18). Further, if two strains are present in a sample, PCR enzymes may switch from one template to another midamplification, generating artificial mosaic sequences (19). A potential solution to these hurdles is to repeat the extraction, amplification, and sequencing process multiple times to determine if the same recombination breakpoint is observed each time, but this approach can be time-consuming and exhaust limited sample volumes. Alternatively, primers can be designed with the aim that that they amplify only one subtype of the virus (14). If a full genome of the recombinant is obtained, its recombinant structure can be characterized using the phylogenetic bootscanning approach (20), which plots the degree to which the target genome groups significantly with one or more reference sequences along the viral genome. This method improves on two-locus phylogenetic analysis by providing an estimate of the recombination breakpoint position.

Next-generation sequencing methods are a promising alternative to traditional sequencing approaches for studying viral genetic diversity. For example, the RNA sequencing (RNA-seq) method by Illumina enables all RNA in a sample to be amplified nonpreferentially after being fragmented, resulting in tens of millions of short sequence reads (21). This technique has the benefit of having no steps that may preferentially amplify one strain of the virus over another, as cDNA synthesis takes place using random primers and can indicate the presence of minor sequence variants in viral quasispecies (22). As RNA sequencing is nonspecific, the vast majority of reads correspond to genetic material from the host or from contaminant species; hence, this method requires substantial computational analysis to extract, assemble, and interpret virus-specific reads (23).

In this study, we applied both traditional PCR and next-generation sequencing methods to two putative recombinants strains that were isolated in Cameroon. We aimed to confirm that these two samples are indeed recombinants and to characterize their mosaic genome structure. In addition, we developed and applied a new computational method, called Deep SimPlot, which uses short-read next-generation sequence data to characterize the structure of viral recombinants.

## MATERIALS AND METHODS

### Study population.

Two putative recombinant HCV strains (EBW034 and EBW436) were obtained during a survey of HCV in Ebolowa, an urban center in southern Cameroon (24). Briefly, individuals were included in the survey if they were age ≥60 years and willing to consent, and they were excluded in the case of dementia or inability to speak a language known to the interviewers. A venous blood sample was obtained from each individual and identified only by a study number (see reference 24 for further details). Of the samples obtained, 252/451 (56%) were HCV seropositive, of which 171 contained detectable HCV RNA. Samples EBW034 and EBW436 were the only recombinants found in this survey, and both were obtained from patients between 65 and 70 years of age. Patient EBW034 had one experience of intravenous injection 30 years ago (antimalarials) and no other past medical history, while patient EBW436 had no experience of intravenous treatments but had received intramuscular injections for the prevention of sleeping sickness and other intramuscular injections for the treatment of yaws, and he was traditionally circumcised with other boys when he was 15 years old. Sample EBW034 had a viral load of 5.2 log IU/ml, while EBW436 had a viral load of 6.6 log IU/ml.

### Sample preparation, RT-PCR, and sequencing.

Samples were first serologically tested for the presence of anti-HCV antibodies using Monolisa anti-HCV Plus version 2 (Bio-Rad). Samples with an optical density-to-cutoff value ratio of ≥6 were then retested with AxSYM version 3 (Abbott Laboratories), with a ratio of ≥20 treated as positive (25). Viral RNA was extracted from positive samples using the QIAamp viral RNA kit (Qiagen). HCV genotyping was performed on a 382-nucleotide (nt) fragment of the NS5B gene amplified in a one-step reverse transcription-PCR (RT-PCR) with SuperScript III (Life Technologies) with primers Pr3 and Pr5, followed by additional amplification with primers Pr4 and Pr5 (see Table 2 for primer details). This result was confirmed by converting RNA to cDNA with AMV-RT (Promega) and random hexamer primers and then amplifying a 360-nt fragment of the core gene with primers CoreOS and CoreOAS and CoreIS and CoreIAS (26). Successful amplicons were sequenced using BigDye Terminator cycle sequencing (Applied Biosystems) and primers Pr3 and Pr5 for the NS5B region and CoreIS and CoreIAS for the core region. Amplicons amplified from RNA extracted from patients EBW034 and EBW436 generated core and NS5B sequences that appeared to be most closely related to different genotypes (specifically, subtypes 4f, 1e, and 1l). In an attempt to confirm recombination and rule out dual infection, the samples were amplified with primers designed to amplify either genotype 1 or 4 only but not both genotypes. Primers were designed using an alignment of all published subtype 4f and genotype 1 genomes (no subtype 1e or 1f genomes were available at the time of analysis). Since the part of the NS5B gene studied was highly variable, we were unable to locate suitable motifs that were well conserved in both genotypes 1 and 4 while also being sufficiently different between the two genotypes. Hence, genotype-specific primers were created for the core region only. These primers (G1-667-Rin-Core and G4-668-Rin-Core, in conjunction with 5′UTR-In-405F) were tested, following a first stage of amplification using the primers 5′UTR-Ex-400F and Gg-767-Rex-Core. For positive controls, these reactions used equivalent volumes of two viral cDNA samples previously identified as belonging to genotypes 1 and 4, with viral loads of between 6.5 and 6.9 log IU/ml.

**TABLE 2:** Details of primers used in this study

Primer name	Sequence	Specificity	Positions^*a*^
5′UTR-Ex-400F	CCTTGTGGTACTGCCTGATAG	Generic	282–299
5′UTR-In-405F	CCTGATAGGGTGCTTGCGAG	Generic	295–311
Gg-767-Rex-Core	CAYGTRAGGGTATCGATGAC	Generic	721–705
G1-667-Rin-Core	GTCABTGGGGCCCCAACTAG	Genotype 1	671–655
G4-668-Rin-Core	ATCATTTGGRCCCCAAGAC	Genotype 4	671–656
NS5B-Ex-Fwd	TGGGGTTCTCRTATGAYACCCGCTGYTTTG	Generic	8248–8274
NS5B-Ex-Rev	AATACCTVGTCATAGCCTCCGTGA	Generic	8637–8617
NS5B-In-Fwd	GAYACCCGCTGYTTTGACTC	Generic	8262–8278
NS5B-In-Rev	TACCTNGTCATAGCCTCCGTGAAGACTC	Generic	8635–8611
CoreOS	ACTGCCTGATAGGGTGCTTGCGAG	Generic	291–311
CoreOAS	ATGTACCCCATGAGGTCGGC	Generic	748–732
CoreIS	AGGTCTCGTAGACCGTGCATC ATG	Generic	324–344
CoreIAS	CAYGTRAGGGTATCGATGAC	Generic	721–705
Pr3	TATGAYACCCGCTGYTTTGCTC	Generic	8259–8278
Pr4	GCNGARTAYCTVGTCATAGCCTC	Generic	8641–8622
Pr5	GCTAGTCATAGCCTCCGT	Generic	8633–8619

a Location numbering is relative to isolate H77 (GenBank accession number AF009606).

### Initial phylogenetic analysis.

An alignment of reference sequences (2) was used to phylogenetically classify the generated sequences. Separate core and NS5B alignments were generated by hand using Se-Al version 2.0 (http://tree.bio.ed.ac.uk), and maximum-likelihood (ML) phylogenies were estimated using the method implemented in GARLI version 0.951 (27). The analysis used a general time-reversible (GTR) nucleotide substitution model, estimated base frequencies, and a gamma distribution model of among-site rate variation. Statistical support for phylogenetic clustering was assessed using an ML bootstrap approach with 500 replicates. Bootstrap scores were summarized using TreeAnnotator (http://beast.bio.ed.ac.uk), and phylogenies were visualized and annotated using FigTree version 1.4 (http://tree.bio.ed.ac.uk).

### Whole-genome Illumina sequencing.

All RNA present in samples EBW034 and EBW436 was sequenced using Illumina RNA-seq next-generation sequencing (28). This method was chosen because (i) the sample volume was too limited to generate whole genomes using primer walking, and (ii) it avoids using HCV-specific primers that might bias which sequences are amplified. Sample processing was performed as described in reference 29. Briefly, sequencing libraries were constructed from 100 ng of total RNA using the NEBNext mRNA sample prep kit 1 (New England BioLabs), according to the manufacturer's guidelines, with minor modifications. This procedure breaks the mRNA into fragments, generates cDNA from the mRNA with random primers, repairs the ends of the cDNA library and appends a d(A) tail, ligates adapters to the ends of the cDNA fragments, and uses PCR to enrich the adapter-ligated cDNA library, resulting in inserts with a median length of 200 nt. The remaining adapter dimers and mRNA were removed using Agencourt AMPure RNAClean XP beads (Beckman Coulter). Amplicons were quantified and assessed for quality using the Quant-IT Qubit double-stranded DNA (dsDNA) high-sensitivity assay (Invitrogen) and 1% E-gel (Invitrogen), respectively, and then sequenced on an Illumina HiSeq 2000, according to standard Illumina protocols, creating 100-nt paired-end reads.

### Genome assembly and analysis.

The sequence reads from Illumina sequencing were extracted using Bam2Fastq and then screened for HCV-related sequences using the BLASTn algorithm (30). Specifically, each pair of reads was compared to a reference alignment of 90 HCV genomes that included all genotypes, and we retained all pairs for which at least one sequence matched one or more HCV reference sequences with an E value ≤0.001. The retained pairs were then assembled into contigs using Velvet (31), with a *k*-mer size of 65. The contigs were assembled into a draft genome using Se-Al 2.0 (tree.bio.ed.ac.uk/software/seal). Last, a completed genome sequence was created by mapping all the HCV-related reads back onto the draft genome using Stampy (32). The resulting BAM file of aligned reads was analyzed with Tablet (33) to confirm assembly quality, and V-Phaser (34) was used to determine which variants represented true biological variants rather than sequencing errors. For sites at which one of these variants was present, the consensus nucleotide was replaced with the appropriate IUPAC ambiguity code (35). The completed genome was then subtyped using the phylogenetic bootscanning approach implemented in the Oxford HCV subtyping tool (36). Bootscanning analysis was performed using a sliding window 400 nt wide that was moved in steps of 50 nt across the viral genome. DIVEIN (37) was used to calculate pairwise maximum-likelihood genetic distances of the HCV genome regions to subtype reference strains under the GTR+Γ nucleotide substitution model.

### Deep SimPlot.

Although phylogenetic bootscanning of the consensus genome is a highly informative and well-validated approach, it does not display all the information generated by a next-generation sequencing run and cannot exclude the possibility that genotype 1 and 4 reads are present across the whole genome (as a result of coinfection) but only at very low numbers in certain regions. Consequently, we devised a new computational tool called the Deep SimPlot method that detects recombination directly from short-read sequence data. We intend this method to be complementary and used in addition to bootscanning of the consensus genome.

First, an alignment of the consensus genome plus two reference genomes (one for each putative ancestor of the recombinant) is constructed. Since the genome assembly procedure (see above) aligns each read against the consensus genome, this positional information can be used to determine the region of the reference genomes to which each read corresponds. Second, the number of nucleotide differences between the read and each of the two reference genomes is calculated. These values are then standardized by converting them into a proportion (i.e., the number of differences between the read and reference X is divided by the total number of differences between the read and references X and Y). This computation is repeated for every read in the sample, and the resulting proportions are plotted against the genomic position of each read, thereby generating a graph of the relative similarity of reads to each reference.

### Nucleotide sequence accession numbers.

The resultant genome and sequences generated by Sanger sequencing and Illumina RNA-seq were uploaded to GenBank and are available under accession numbers KR870888 to KR870894.

## RESULTS

### Initial subtyping.

**FIG 1:**
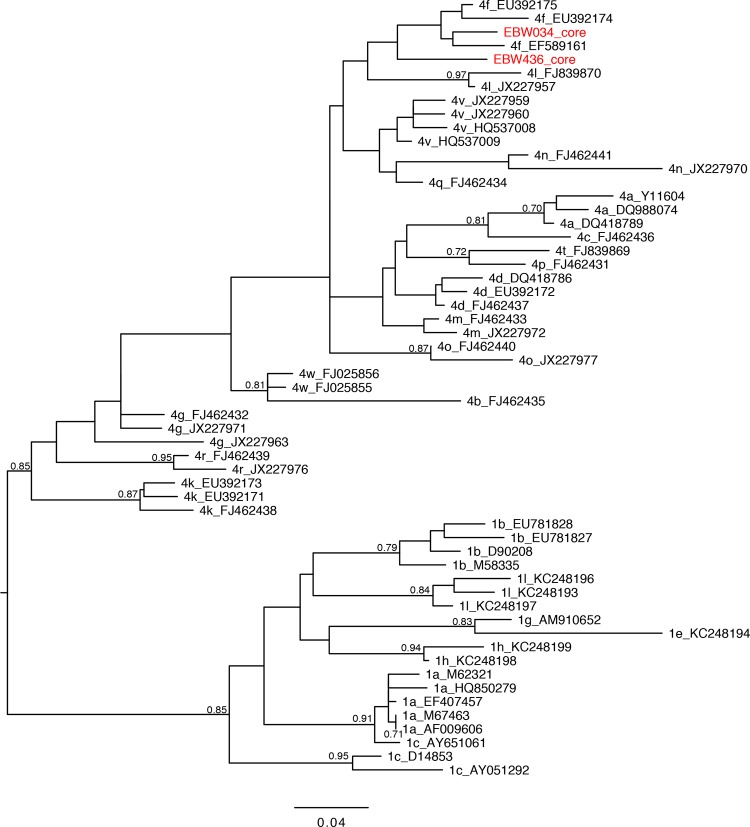
Estimated maximum-likelihood midpoint-rooted phylogeny of subgenomic core sequences. Nodes with bootstrap support of >70% are labeled with their bootstrap support values. The sequences generated in this study are highlighted in red. The branch lengths are in units of expected substitutions per site (see scale bar). Reference sequences are labeled with their subtype and accession number.

**FIG 2:**
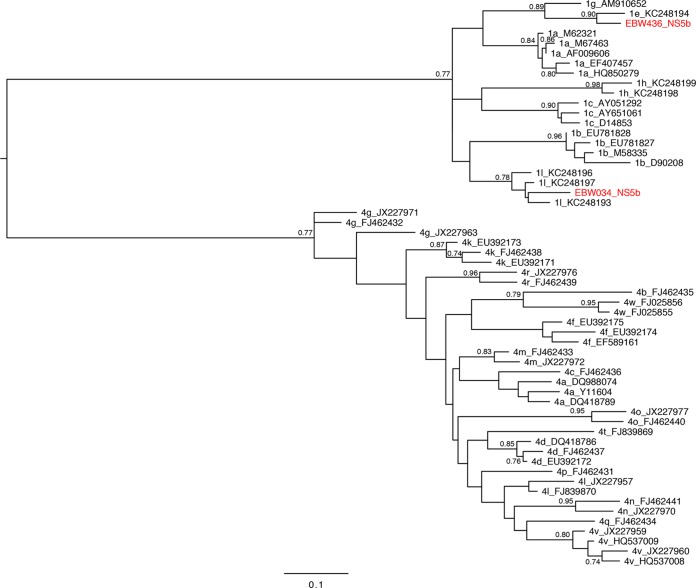
Estimated maximum-likelihood midpoint-rooted phylogeny of subgenomic NS5B sequences. See Fig. 1 legend for further details.

Figures 1 and 2 show the maximum-likelihood trees estimated from the core and NS5B alignments, respectively. These trees include reference strains plus the subgenomic sequences obtained using RT-PCR from two putative recombinant samples (EBW034 and EBW436). In the core phylogeny, EBW034 and EBW436 both fall within subtype 4f (Fig. 1), while in the NS5B phylogeny, EBW034 falls within subtype 1l, and EBW436 is closest to subtype 1e (Fig. 2). The subtype assignment of EBW034 and EBW436 in the core phylogeny has low bootstrap support, due to the limited genetic variation in that subgenomic region, although there is strong support for the placement of both within genotype 4. In contrast, in the NS5B region, both samples are assigned to specific subtypes with high bootstrap scores. In addition, the BigDye sequencing data show no evidence of consistent minor peaks (<2% of peaks had minor variants), implying that dual infection is unlikely. These results are consistent with, but do not confirm, the hypothesis that both samples are recombinant.

### Genotype-specific PCR amplification.

The genotype 1-specific primers amplified the genotype 1 control only, while the genotype 4-specific primers were able to amplify the genotype 4 control and both EBW34 and EBW436. Figure S1 in the supplemental material shows a phylogeny of the sequences obtained from the genotype 4-specific amplification of the core region alongside G1 and G4 reference genomes and the EBW34 and EBW436 core sequences obtained from initial subtyping, demonstrating that the sequences generated from subtype-specific amplification match those generated from the earlier assay. Genotype-specific amplification was not attempted for NS5B, because no suitable primers in this subgenomic region were apparent (see Materials and Methods for details).

### Whole-genome Illumina sequencing.

Illumina sequencing generated 9,861,538 reads indexed to sample EBW436, each 100 nt long. Of these, 3,084 (0.00031%) reads exhibited significant similarity to a panel of HCV reference genomes. Using Velvet, the reads were assembled into 12 contigs that provided complete coverage of the EBW436 genome. The contigs ranged in length from 66 nt to 2,958 nt, and coverage ranged from 1 to 62 reads per site, with an average coverage of 28.1 reads per site. When all EBW436 reads were mapped back to the draft genome created from the contigs, 85 single nucleotide polymorphisms (SNPs) were observed, and no read diverged from the consensus sequence at more than one position.

**FIG 3:**
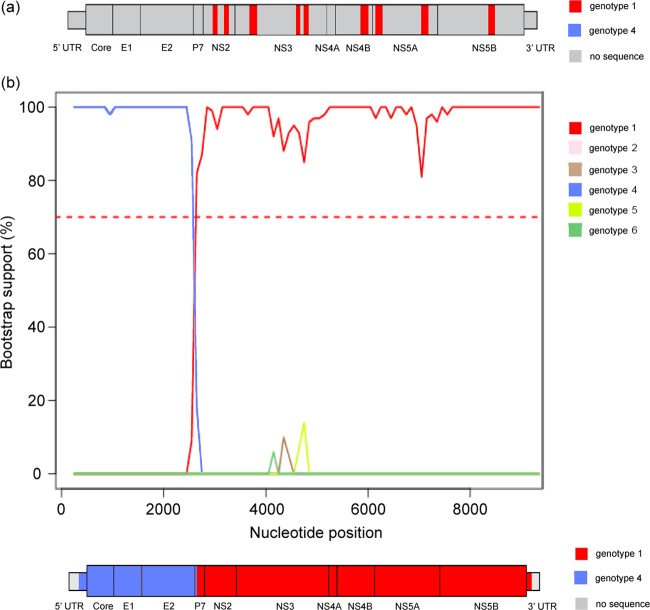
(a) Schematic of the RNA-seq data obtained for sample EBW034. A total of 9 genome regions were assembled from 14 reads and are colored in red. All regions showed the closest sequence similarity to subtype 1l. (b) Bootscan plot of the EBW436 consensus genome, generated using the Oxford HCV automated subtyping tool. Top, bootscan plot, which displays the bootstrap support for the clustering of EBW436 with genotype reference genomes in 400-nt sliding windows across the viral genome. The curves are colored according to the genotype key on the right. Bottom, schematic of the RNA-seq consensus genome obtained for sample EBW436. Genome regions with an association to a particular genotype supported by >70% bootstrap support (also shown at top as a red horizontal dashed line) are colored according to the genotype in question.

Illumina sequencing generated 5,415,510 reads indexed to sample EBW034, but only 14 (0.0000026%) of these exhibited significant similarity to a panel of HCV reference genomes. Assembly of these reads using Velvet was not possible due lack of read overlap. The 14 reads covered approximately 13% of the EBW034 genome (totaling 1,400 nt). Figure 3a shows the location of each read in the HCV genome; reads were observed in the NS2, NS3, NS4B, NS5A, and NS5B genes. The pairwise genetic distances between each read and a panel of HCV reference strains were calculated using DIVEIN (37). In every case, subtype 1l was closest to the EBW034 reads. Further phylogenetic analysis of these reads was not undertaken due to their short lengths.

Figure 3b shows the result of the bootscanning analysis applied to the consensus genome obtained for EBW436. There is strong statistical support for a mosaic genome structure: from the 5′ untranslated region (UTR) to the start of P7, the EBW436 genome groups with genotype 4 and then quickly switches to grouping with genotype 1 until the 3′ end of the genome. The analysis indicates that the recombination breakpoint is between positions 2450 to 2750 nt (relative to H77), near the 5′ end of the P7 gene.

### Deep SimPlot analysis.

To directly visualize the short-read sequence data and rule out the possibility that genotypes 1 and 4 were both present in the sample, we developed a new graphical approach for detecting viral recombination called Deep SimPlot (see Materials and Methods). This method was applied to the 3,084 HCV reads obtained from sample EBW436 (too few reads from sample EBW034 were available for analysis). The method first requires the selection of two reference genomes. Since the partial NS5B sequence of EBW436 unambiguously grouped with subtype 1e (Fig. 2), a reference genome belonging to that subtype was chosen (GenBank accession no. KC248194). However, it was more difficult to select an optimal genotype 4 reference strain. Although the partial core sequence of EBW436 (Fig. 1) grouped phylogenetically with subtype 4f, bootstrap support for that grouping was weak. Analysis of pairwise genetic distances between the core region of the EBW436 consensus genome and a panel of reference strains showed that EBW436 had the shortest genetic distance to genomes from subtype 4q, not subtype 4f. This was supported by a maximum-likelihood tree of this same alignment (see Fig. S2 in the supplemental material). To accommodate this uncertainty, we therefore performed the Deep SimPlot method twice, once using subtypes 4f and 1e as reference genomes, and once using subtypes 4q and 1e.

**FIG 4:**
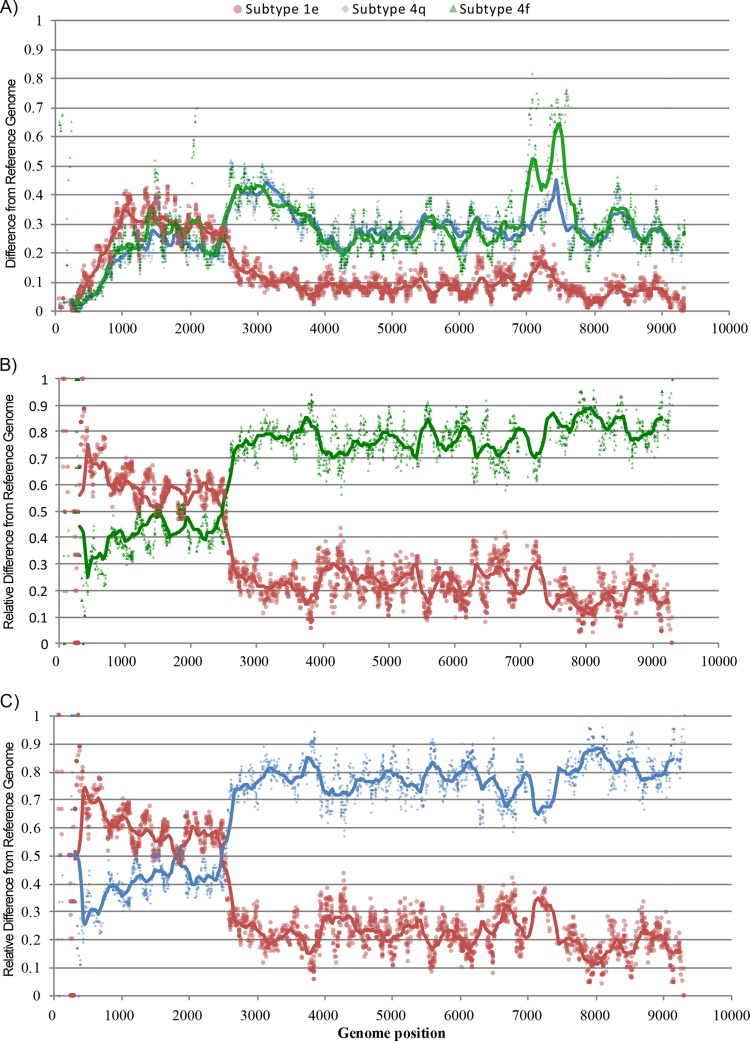
Deep SimPlot analysis of the mapped reads from sample EBW436 compared to three reference genomes: subtype 1e (red), subtype 4f (green), and subtype 4q (blue). (a) Proportion of sites that differ between each read and reference genome (*p*-distance). Markers are colored according to the appropriate reference genome. The curves represent a sliding average of the points within a 500-nt window. (b and c) Relative difference of each read to two reference genomes, subtypes 1e and 4f (b), and subtypes 1e and 4q (c).

The results of the Deep SimPlot analysis are shown in Fig. 4. Figure 4a shows the absolute genetic difference between each EBW436 read and the three reference genomes, whereas Fig. 4b and c show the relative genetic distance of each read to two reference genomes. Figures 4b and c provide strong support for recombination and clearly indicate a recombination breakpoint around position 2530 nt (corresponding to position 2645 in H77), which agrees with the bootscanning analysis of the consensus genome (Fig. 3). The results are robust to the particular genotype 4 reference used (subtype 4f in Fig. 4b and subtype 4q in Fig. 4c). Before the breakpoint, only 10.7% of the reads are closer to subtype 1e than to 4q (Fig. 4c), and only 13% of the reads are closer to subtype 1e than 4f (Fig. 4b). After the breakpoint, no reads are closer to genotype 4 than genotype 1. This difference between genome regions likely results from low sequence variation in the 5′ UTR and core regions (see Fig. 4a), such that just one or two mutations may make a genotype 4 read appear more similar to the genotype 1 reference. The Deep SimPlot analysis is capable of ruling out the hypothesis of dual infection; if the sample was dually infected, no breakpoint would be seen, and all genome regions should exhibit at least some reads that are genotype 1-like and some that are genotype 4-like.

## DISCUSSION

The first goal of this study was to confirm recombination in the two HCV samples, EBW436 and EBW034. No results were inconsistent with the hypothesis of recombination for either sample, and the mosaic genome structure of EBW436 was proven by analysis of reads from non-HCV-specific next-generation sequencing (Illumina RNA-seq). Although a complete genome could not be assembled for sample EBW034, the small number of reads that were obtained were all classified as subtype 1l, consistent with the results of the subgenomic PCR approach (Fig. 2). The low coverage of EBW034 may be in part due to the lower viral load of this sample. Unfortunately, no further patient material was available to repeat the RNA-seq approach for sample EBW034.

The parental lineages of these two samples belong to genotypes 1 and 4. HCV is highly prevalent in Cameroon (24, 25, 38), and the presence of both genotypes in the country is well established (39). In a previous study of HCV genetic diversity in Cameroon, 45% of the isolates were classified as genotype 1 and 31% as genotype 4 (40). Of those genotype 1 isolates, 34% belonged to subtype 1e and 33% to subtype 1l, which are precisely the two subtypes detected in the two samples investigated here. While we can be confident that the second parental lineage of sample EBW436 belongs to genotype 4, we are less sure as to which subtype that parent might belong. The partial core sequence of EBW436 groups with subtype 4f (with low bootstrap support; Fig. 1) but has a slightly smaller genetic distance to subtype 4q strains. Subtype 4f is highly prevalent in Cameroon, accounting for 60% of all genotype 4 infections in a previous survey (41). Subtype 4q is less common and has been reported in several sub-Saharan African countries, including Rwanda, Burundi, and the Democratic Republic of Congo (42). It is possible that the true genotype 4 parent of sample EBW436 is a divergent strain that falls outside currently defined subtypes. In summary, the high prevalence of HCV in southern Cameroon and the cocirculation there of subtypes 1e and 1l and various subtypes of genotype 4 have likely increased the chances of HCV coinfection, resulting in the creation of the viable recombinants observed in this study. Neither patient had a medical history containing risk factors for dual infection (e.g., injectable-drug use [43]), making it less likely that recombination occurred in the patients and thus implying that these recombinants are CRFs circulating in Cameroon.

Our results follow the pattern seen in the literature on HCV breakpoint locations: intergenotypic breakpoints tend to occur around the boundary between the structural and nonstructural genes of HCV (Table 1), and the EBW436 breakpoint occurs in the small P7 gene. It is possible that viable breakpoints for intergenotypic recombination vary according to the genotypes involved; HCV has an RNA secondary structure that is sensitive to mutation and may impact virus viability (44). Further analysis of HCV recombination breakpoint positions may help understand what factors determine viable breakpoint placement, as has been undertaken for HIV (45).

We also sought to investigate different molecular methods of detecting HCV recombination versus the null hypothesis of dual infection. Four different approaches were attempted: (i) Sanger sequencing of two subgenomic regions with standard primers, (ii) Sanger sequencing using subtype-specific primers, (iii) bootscanning analysis of a consensus genome assembled from RNA-seq-generated reads, and (iv) Deep SimPlot analysis of the RNA-seq-generated reads. Methods iii and iv were complementary and together established the mosaic nature of the genome of sample EBW436 and the location of its recombination breakpoint. The tests performed in this study go beyond those often employed when HCV recombination is reported in the literature. For example, recombination has been proposed on the basis of discordance between subgenomic sequences obtained from the E1 and NS5B regions (46). It is debatable whether this constitutes sufficient evidence to conclude that a sample contains a recombinant virus. In many applications, amplification and sequencing of the core and NS5B regions, followed by confirmatory sequencing of clonally isolated strands, should be sufficient. However, if in the future, HCV recombination becomes more clinically relevant (for example, if direct-acting antiviral treatment responses vary among genotypes), the accurate detection of recombination without the risk of false positives will become essential.

One of the difficulties in characterizing highly varied RNA virus genomes is the possibility that screening techniques, particularly PCR, will be biased toward some viral variants over others. We attempted to mitigate this problem through the use of Illumina RNA-seq. No RNA was removed or filtered during sample preparation, and although the vast majority of the sequence generated was not HCV related, the coverage of the EBW436 genome was sufficient to assemble a reliable consensus genome. The BLAST-based algorithm used here to identify HCV-related reads took ∼130 h to process the 9.8 million reads for sample EBW436; therefore, the optimization of this procedure in the future would be beneficial. However, only 14 of the 5.4 million reads generated from sample EBW034 were categorized as HCV, despite the comparative leniency of the BLAST-based algorithm used. Thus, RNA-seq without HCV-specific amplification or any attempt to enrich for viral RNA is unlikely to be repeatedly reliable for samples with low viral loads or those which have been repeatedly frozen and thawed.

The Deep SimPlot approach developed here and presented in Fig. 4 provides an intuitive visual representation of the RNA-seq read data and displays the mosaic structure of a recombinant genome in a manner analogous to the bootscanning method applied to the consensus genome (Fig. 3), all while retaining the information provided by the individual reads and thus discriminating between dual infection and recombination. Further investigation of the Deep SimPlot technique will help elucidate its requirements, statistical power, and limitations. Important questions for future research include (i) how the best reference genomes should be selected for analysis, and (ii) whether the analysis can or should be performed with multiple reference genomes.

## Supplementary Material

Supplemental material
