# Supplementary material for: Characterization of Hepatitis C Virus Recombination in Cameroon by Use of Nonspecific Next-Generation Sequencing
Source: J Clin Microbiol. 2015 Sep 16;53(10):3155–64. doi: 10.1128/JCM.00483-15 (PMC4572555; doi:10.1128/JCM.00483-15)
Supplement: Supplemental material [file supp_53_10_3155__index.html]

Characterization of Hepatitis C Virus Recombination in Cameroon by Use of Nonspecific Next-Generation Sequencing — Supplemental material 

# Characterization of Hepatitis C Virus Recombination in Cameroon by Use of Nonspecific Next-Generation Sequencing

## Supplemental material

- Supplemental file 1 -

  Fig. S1 (Estimated maximum likelihood midpoint-rooted phylogeny of subgenomic core sequences) and S2 (Estimated maximum likelihood midpoint-rooted phylogeny of the core section of the generated EBW436 consensus genome, along with reference sequences)

  PDF, 100K
