# Supplementary material for: Characterization of Hepatitis C Virus Recombination in Cameroon by Use of Nonspecific Next-Generation Sequencing
Source: J Clin Microbiol. 2015 Sep 16;53(10):3155–64. doi: 10.1128/JCM.00483-15 (PMC4572555; doi:10.1128/JCM.00483-15)
Supplement: Supplemental material [file JCM.00483-15_zjm999094494so1.pdf]

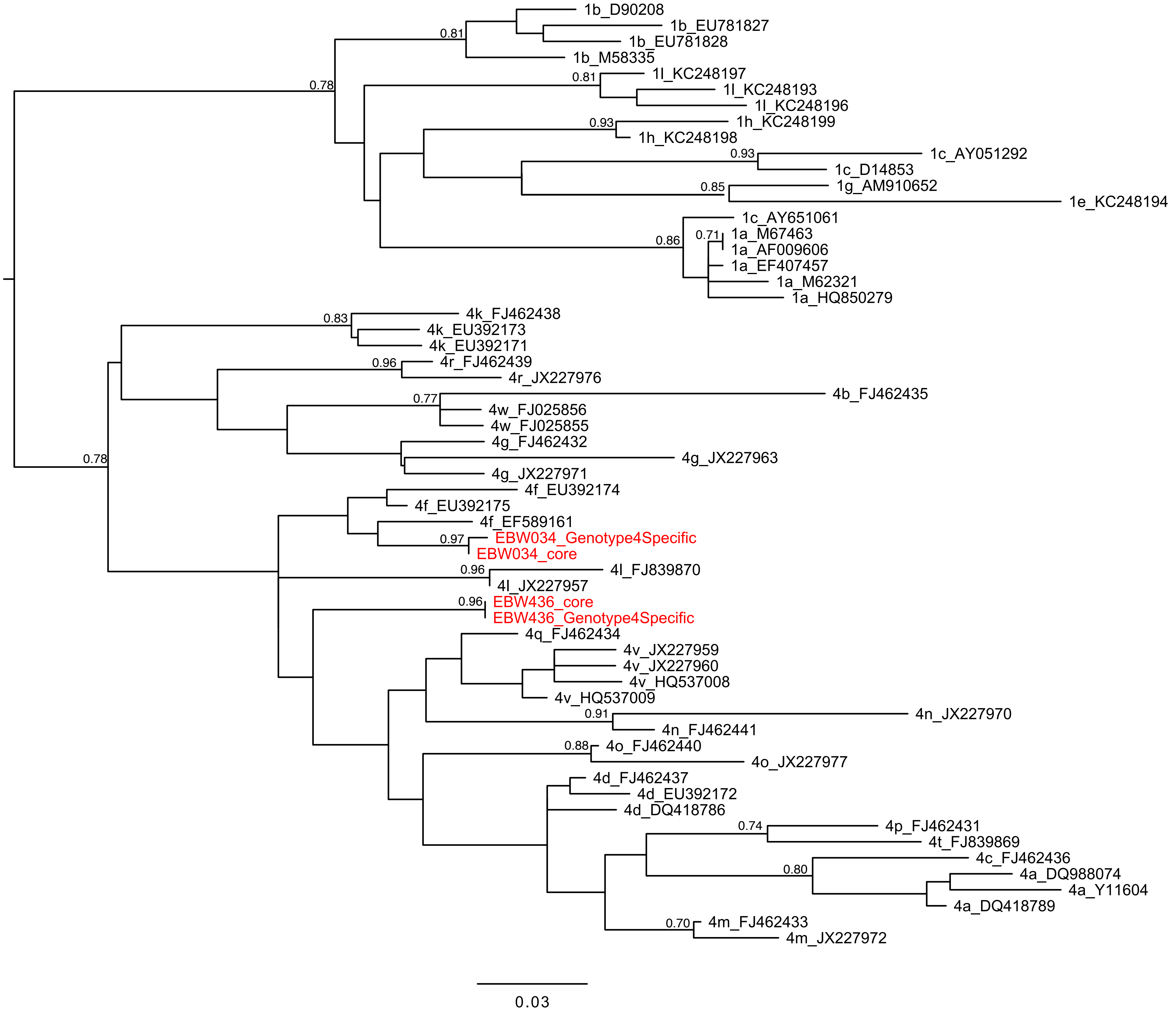
1b_D90208
1b_EU781827
1b_EU781828
1b_M58335
1l_KC248197
1l_KC248193
1l_KC248196
1h_KC248199
1h_KC248198
1c_AY051292
1c_D14853
1g_AM910652
1e_KC248194
1c_AY651061
1a_M67463
1a_AF009606
1a_EF407457
1a_M62321
1a_HQ850279
4k_FJ462438
4k_EU392173
4k_EU392171
4r_FJ462439
4r_JX227976
4b_FJ462435
4w_FJ025856
4w_FJ025855
4g_FJ462432
4g_JX227963
4g_JX227971
4f_EU392174
4f_EU392175
4f_EF589161
EBW034_Genotype4Specific
EBW034_core
4l_FJ839870
4l_JX227957
EBW436_core
EBW436_Genotype4Specific
4q_FJ462434
4v_JX227959
4v_JX227960
4v_HQ537008
4v_HQ537009
4n_JX227970
4n_FJ462441
4o_FJ462440
4o_JX227977
4d_FJ462437
4d_EU392172
4d_DQ418786
4p_FJ462431
4t_FJ839869
4c_FJ462436
4a_DQ988074
4a_Y11604
4a_DQ418789
4m_FJ462433
4m_JX227972
0.03

Figure S1

Estimated maximum-likelihood midpoint-rooted phylogeny of sub- genomic Core sequences. Nodes with bootstrap support >70% are labelled with their bootstrap support. Sequences generated in this study are highlighted in red. Branch lengths are in units of expected substitutions per site (see scale bar). Reference sequences are labelled with subtype and accession number. Samples EBW034_core and EBW436_core are the product of initial subtyping (see Figure 1), while samples EBW034_Genotype4Specific and EBW436_Genotype4Specific are the product of subtype-specific amplification.

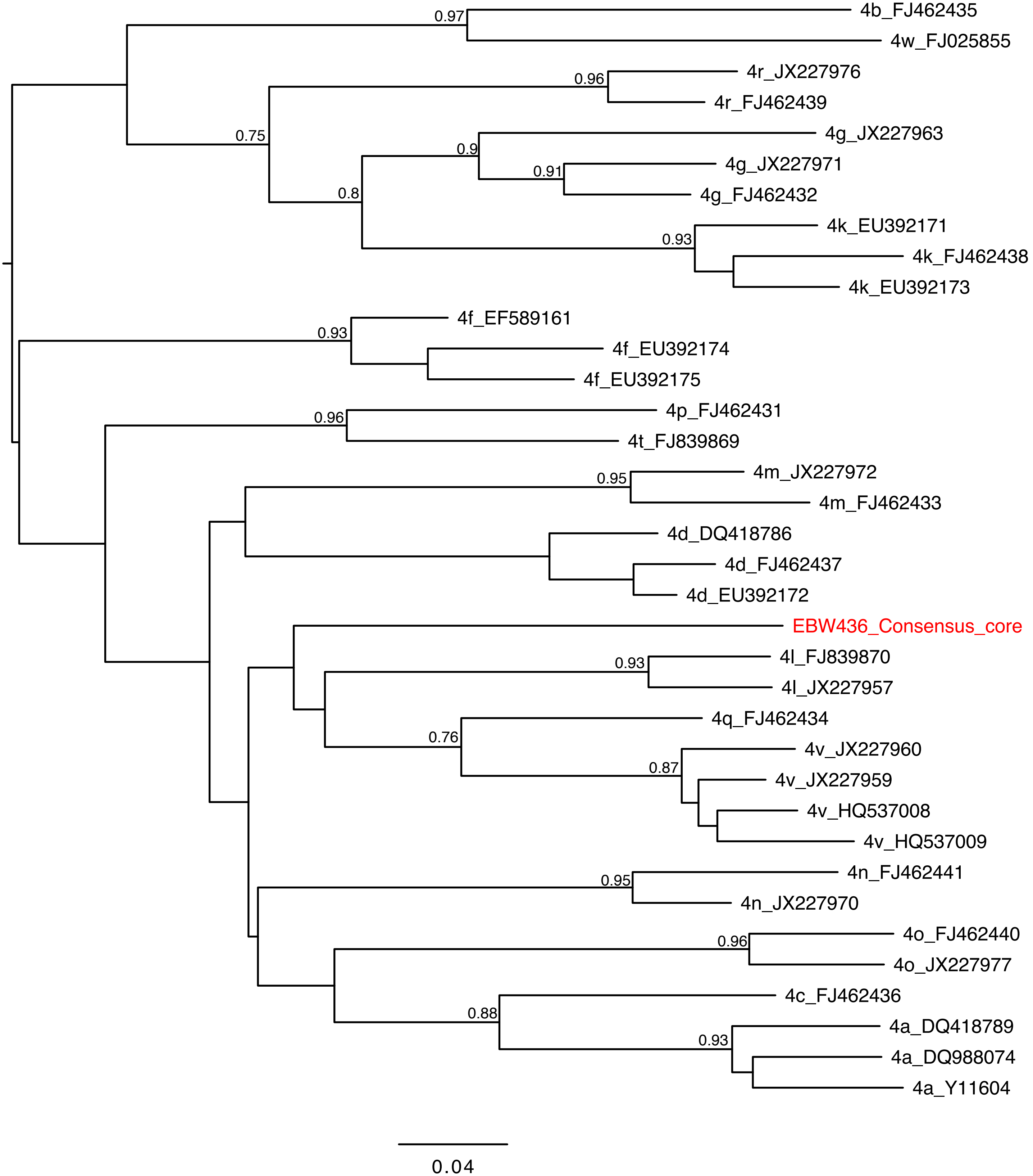

0.97
4b_FJ462435
4w_FJ025855
0.96
4r_JX227976
4r_FJ462439
0.75
0.9
4g_JX227963
0.91
4g_JX227971
4g_FJ462432
0.8
0.93
4k_EU392171
4k_FJ462438
4k_EU392173
0.93
4f_EF589161
4f_EU392174
4f_EU392175
0.96
4p_FJ462431
4t_FJ839869
0.95
4m_JX227972
4m_FJ462433
4d_DQ418786
4d_FJ462437
4d_EU392172
EBW436_Consensus_core
0.93
4l_FJ839870
4l_JX227957
4q_FJ462434
0.76
0.87
4v_JX227960
4v_JX227959
4v_HQ537008
4v_HQ537009
0.95
4n_FJ462441
4n_JX227970
0.96
4o_FJ462440
4o_JX227977
4c_FJ462436
0.88
0.93
4a_DQ418789
4a_DQ988074
4a_Y11604
0.04

Figure S2

Estimated maximum-likelihood midpoint-rooted phylogeny of the core section of the generated EBW436 consensus genome, highlighted in red, along with reference sequences. Nodes with bootstrap support >70% are labelled with their bootstrap support. Branch lengths are in units of expected substitutions per site (see scale bar). Reference sequences are labelled with subtype and accession number.
